# Salivary Diagnostics in Pediatrics: Applicability, Translatability, and Limitations

**DOI:** 10.3389/fpubh.2017.00083

**Published:** 2017-04-20

**Authors:** Mona Hassaneen, Jill L. Maron

**Affiliations:** ^1^Mother Infant Research Institute at Tufts Medical Center, Boston, MA, USA; ^2^Division of Newborn Medicine, Floating Hospital for Children at Tufts Medical Center, Boston, MA, USA

**Keywords:** saliva, diagnostics, pediatrics, newborns, genetics

## Abstract

In the last decade, technological advances, combined with an improved appreciation of the ability of saliva to inform caregivers about both oral health and systemic disease, have led to the emergence of salivary diagnostic platforms. However, the majority of these assays have targeted diseases that more commonly affect the adult population, largely neglecting infants and children who arguably could benefit the most from non-invasive assessment tools for health monitoring. Gaining access into development, infection, and disease through comprehensive “omic” analyses of saliva could significantly improve care and enhance health access. In this review, we will highlight novel applications of salivary diagnostics in pediatrics across the “omic” spectrum, including at the genomic, transcriptomic, proteomic, microbiomic, and metabolomic level. The challenges to implementing salivary platforms into care, including the effects of age, diet, and developmental stage on salivary components, will be reviewed. Ultimately, large-scale, multicenter trials must be performed to establish normative biomarker values across the age spectrum to accurately discriminate between health and disease. Only then can salivary diagnostics truly translate into pediatric care.

## Introduction

**Figure 1:**
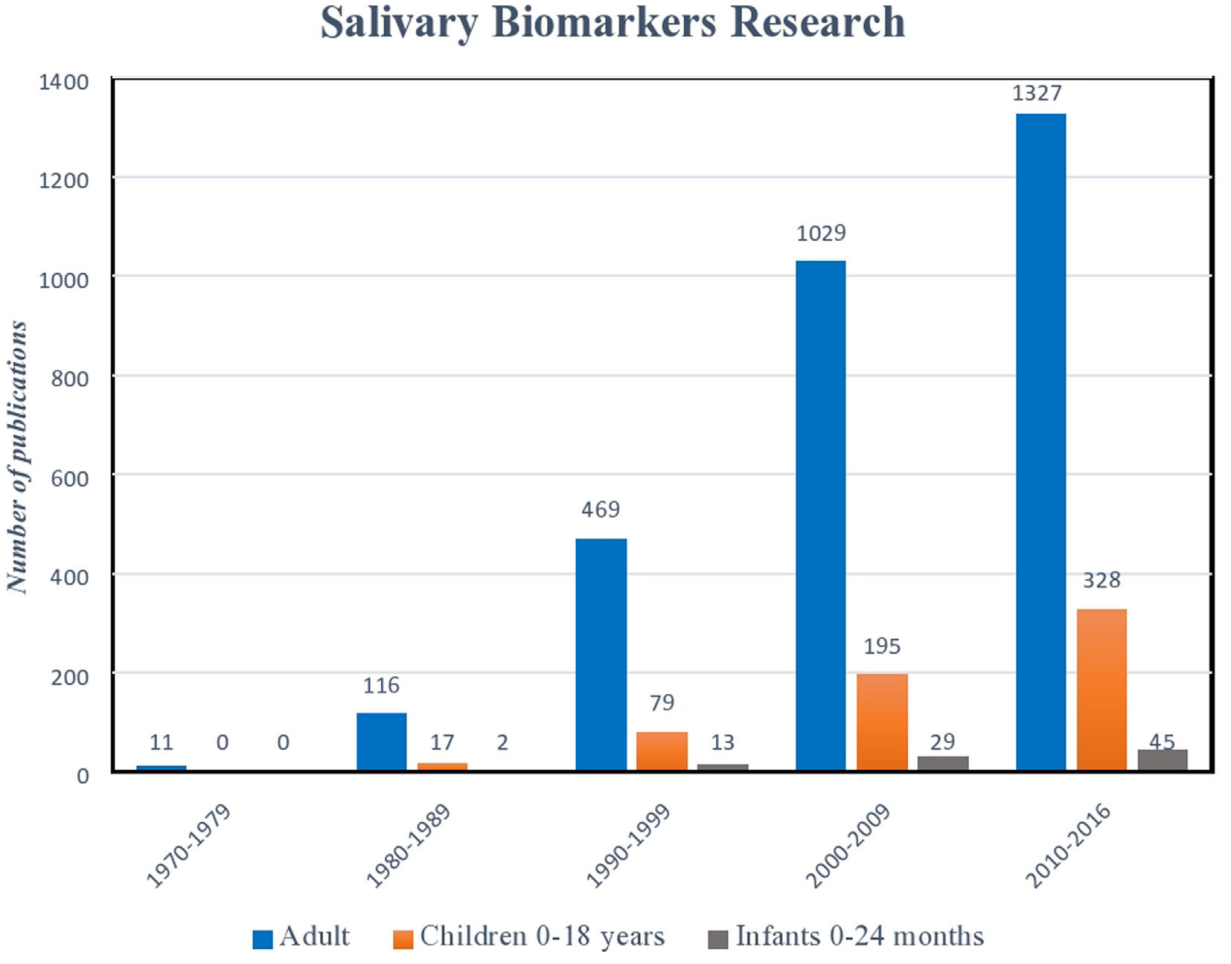
**Histogram illustrating exponential growth of salivary diagnostic platforms over time**. Though the number of assays designed for children and infants has also increased, there remains a relative paucity of platforms targeted for this age demographic. (Data derived from a PubMed search 9.9.16: keywords “salivary biomarkers”; limits: humans.)

Serum biomarkers have long been the gold standard for diagnostic testing (1–6). However, in recent years, advances in biotechnology, combined with a clinical demand for more user-friendly and non-invasive platforms, have led to the emergence of salivary diagnostic assays to better monitor disease, infection, and development (7–11). Perhaps, no other patient population could benefit more from these advances than pediatrics. The avoidance of serial phlebotomy for monitoring our most at-risk patients reduces trauma and limits anemia (12, 13). Further, as national and international organizations, such as the United States Food and Drug Association (USFDA) now mandate enrollment of children in clinical trials, assays that do not rely on invasive blood sampling offer a safer, more appealing alternative (14–16). While the benefits of salivary analysis in the pediatric population are plentiful, translating assays into clinical care remains a challenge. Salivary assay development for the adult population has seen exponential growth in recent decades, while diagnostics that aim at the unique diseases and conditions affecting infants and children lag significantly behind (Figure 1). Defining the clinical significance of individual variations in biomarker levels, determining thresholds that clearly discriminate between health and disease, and understanding the impact of age, diet, and development on the composition of saliva present hurdles to implementation. Nevertheless, a wealth of information can be gained from a mere drop of human saliva. From predicting physiological development and biological functions, to microbial and metabolic analyses, saliva is providing pediatric caregivers and researchers with an exciting new tool for exploration (9, 17–21).

In this review, we will highlight novel applications of salivary analyses across the “omic” spectrum, including at the genomic, transcriptomic, proteomic, metabolomic, and microbiomic level. The important impact of age and development on saliva components will be reviewed, and specific attention will be given to emerging platforms for use in both neonatal and pediatric populations. Limitations to assay development and clinical implementation will be discussed to further our understanding of both the applicability and the translatability of salivary diagnostics in the pediatric population.

## Salivary Variation Across the Pediatric Age Spectrum

**Figure 2:**
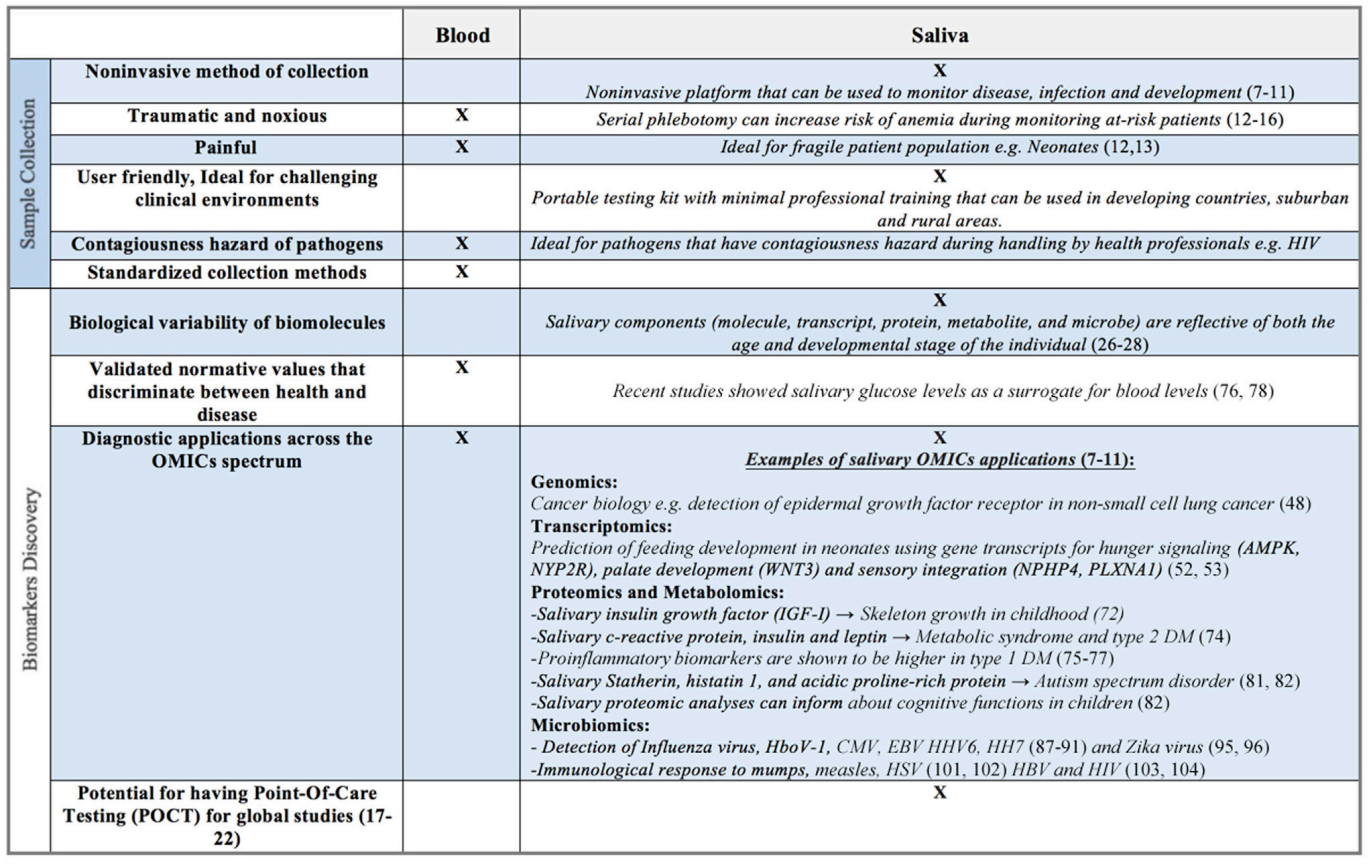
**Benefits and limitations of saliva as a biofluid for biomarker discovery compared to serum, plasma, or whole blood**.

Though largely composed of water, human saliva contains electrolytes, microorganisms, enzymes, proteins, immunoglobulins, nucleic acids, enzymes, hormones, mucins, and drugs (22–24). Commercially available collection and stabilizing kits (e.g., DNA Genotek, Oasis Diagnostics^®^) allow for both ease of collection and stabilization of constituents, often for weeks at a time at room temperature. These components, whether molecule, transcript, protein, metabolite, or microbe, are reflective of both the age and developmental stage of the individual (25–27). For example, salivary enzymes, such as amylase, are known to increase from early infancy through adolescence, ultimately peaking in adulthood (28–31). Salivary electrolyte levels are also known to vary with age. Calcium and magnesium are significantly higher in infancy compared to later in life; sodium to potassium ratios reach their highest levels in adolescence, likely corresponding to aldosterone surges associated with puberty (22). Such analyte concentration variability presents both opportunities and challenges for the investigator. The ability to non-invasively monitor growth and development, in real time, provides great promise (Figure 2). However, an investigator must be aware of biological changes that occur with age, as well as unique patient populations and situations, which may directly affect the oral cavity and its constituents, ultimately impacting the reliability and applicability of salivary assays.

There are critical environmental and developmental changes that take place in early life that also have a direct impact on saliva. For infants born prematurely (<37 weeks’ gestation), not only may the ongoing development of salivary glands impact filtration, secretion, and diffusion of molecules into saliva (32) but also mode of birth (cesarean vs. vaginal), initiation of feeds, and type of nutrition (breast milk vs. formula), may directly affect microbial colonization and diversification in the oral cavity. In recent years, multiple investigators have begun to describe and highlight the rapid microbial colonization of the human shortly after birth (33–35). Deviations from normal deliveries, delays in the initiation of enteral nutrition, and prolonged hospitalizations are now known to significantly impact colonization of the gastrointestinal (GI) system (36–38). As the entry point to the GI system and a critical player in overall GI health, saliva undoubtedly is equally affected by these variables. In addition, studies have shown a significant increase in oral microbial colonization and taxa variability as an infant grows and develops (39). Teeth eruption during the first few months to years of life and exposure to solid foods are considered to be the major contributors to this variability (39, 40). Oral bacteria that reside in gingival crevices and around teeth begin to emerge with tooth eruption. In parallel, both albumin and immunoglobulin (Ig) G levels rise in saliva with increasing mucosal permeability (41). Further, studies have shown that with the introduction of solid foods, salivary peptidomal profiles are altered (39) and Ig levels shift from non-specific innate immunity to specific immune response elements, e.g., IgA and β-2 microglobulin of MHC class I. These alterations are believed to reflect the development of adaptive immune responses after exposure to different noxious dietary and environmental substances (39, 40). These age-specific differences remind investigators that they must consider the developmental stage of an individual when designing and importantly, interpreting salivary assays. Normative values and microbial diversification will vary with age, and assay interpretation must reflect these findings appropriately.

## Salivary “Omics”

### Salivary Genomics

The oral cavity, though not saliva in particular, has offered caregivers a direct, non-invasive source of genomic material. In recent years, genetic testing has moved away from reliance upon invasive blood sampling toward user-friendly buccal swabs (42, 43). From commercially available at-home genetic testing kits (e.g., http://23andme.com, http://Ancestry.com) to paternity testing in the NICU, the ease with which sufficient quality and quantity of DNA samples can be obtained has markedly improved (43). However, beyond cellular DNA which is best harvested through cells, saliva is likely a rich source of cell-free and exosomal DNA that may provide caregivers with specific opportunities to monitor the overall health of the individual and further explore cell-to-cell communication (44–46). While research in this area continues to emerge, it is easy to see its applicability to child health. Genomic analysis of plasma cell-free DNA is directly impacting the field of cancer biology (47). Oncologists may now monitor the genomes of cell-free tumor DNA in plasma samples in order to generate targeted and personal therapies, assess drug resistance, and monitor a patient’s response to therapy by quantitative analysis of tumor load. Analysis of salivary cell-free DNA may provide similar insight and be highly beneficial in children affected by cancer who are already undergoing countless needle sticks and invasive procedures. Indeed, Pu et al. have recently demonstrated the applicability of a salivary assay for the detection of a genomic deletion in the epidermal growth factor receptor in patients with non-small cell lung cancer (48). While future studies are needed to demonstrate the role of cell-free salivary DNA in child health, it nevertheless offers yet another opportunity to improve delivery of care in this vulnerable population.

### Salivary Transcriptomics

Our laboratory was one of the first to publish real-time developmental information available at a transcriptomic (RNA or gene expression) level in the newborn (49–51). In our original article, we demonstrated that transcripts, indicative of all major organ systems, were readily detected in an infant’s mouth (49). Genes identified were known to play a role in the developing GI, nervous, and hematological systems. While the trafficking mechanisms of these gene transcripts remain largely unknown, these initial hypothesis discovery experiments led to a series of targeted assays aimed at better defining developmental milestones and phenotypes.

Using high-throughput screening tools, such as multiplexed reverse transcriptase-quantitative polymerase chain reaction, on total RNA extracted from as little as 5 µL of neonatal saliva, we have been able to identify a panel of genes whose combined expression profiles may help neonatal caregivers to objectively assess oral feeding skills (52, 53). Genes identified on the panel are involved in diverse biological functions including hunger signaling (*AMPK, NYP2R*), palate development (*WNT3*), and sensory integration (*NPHP4, PLXNA1*). In initial studies, the combined salivary expression profile of these biomarkers was shown to be up to 78% accurate in predicting mature oral feeding skills in the newborn. In addition, our laboratory has been the first to link expression levels of a well-described speech–language gene, *FOXP2*, to oral feeding success in the newborn (54, 55). These experiments have laid the foundation for future studies to non-invasively explore developmental biology in our youngest patients and offer caregivers an enormous opportunity to utilize salivary transcriptomics to further explore, diagnose, and potentially prevent other areas of neonatal pathology where disrupted development results in unique and often life-threatening diseases including bronchopulmonary dysplasia, necrotizing enterocolitis (NEC), or retinopathy of prematurity. However, as a newly emerging field, it is important to recognize the potential impact of growth and biology on assay applicability. As stated previously, defining normative values across the age spectrum, exploring sex differences in expression patterns, understanding the role of salivary gland development, microbial colonization patterns, diet, and tooth eruption on gene expression will take prospective, collaborative, multicenter trials. Failing to perform the necessary experiments, such as observational studies to examine developing microbial colonization patterns or to establish normative reference genes for appropriate gene expression analyses over time, will directly impact our ability to translate these exciting discoveries to the bedside.

### Salivary Proteomics and Metabolomics

Saliva has been estimated to contain approximately 2,000 peptides, comprising 40–50% of total secreted body proteins (56). Unlike nucleic acids (DNA and RNA) that traditionally require the additional step of extraction prior to analysis, salivary proteins may be detected and quantified directly after collection. This ease of processing, combined with their relative stability compared to either DNA or RNA, makes proteins ideal biomarkers. Recent advances in technology and bioinformatics has allowed for the comprehensive profiling of hundreds to thousands of proteins from a single sample source to improve our understanding of the physiological, as well as the pathological, status of the human being (57–61).

To date, salivary protein biomarkers have been described for multiple adult oral and systemic diseases, including breast, pancreatic, and oral cancers (62–65), as well as autoimmune diseases, such as Sjögren’s disease, diffuse systemic sclerosis, rheumatoid arthritis, and systemic lupus erythematosus (SLE) (66). Moreover, studies have investigated the role of salivary proteomic analyses to predict myocardial infarction, diabetes mellitus types 1 and 2, and pulmonary diseases (67–69). While the pediatric patient population is ripe for similar diagnostic advances, here too, an investigator must pay specific attention to unique circumstances, including ongoing and rapid development as well as hormonal changes associated with puberty, which may impact proteomic analyses in newborns, infants, and children.

The salivary proteome varies from childhood to adolescence and is often dependent upon growth (70–72). For example, concentrations of salivary insulin growth factor (*IGF-I*) may vary and serve as an indicator of skeletal growth throughout childhood (71). Nutritional status of the child also affects salivary biomarkers. Malnourished children have specific salivary proteomic variations associated with protein energy under nutrition or PEU (73), and children affected with type 1 diabetes have been shown to have higher levels of salivary pro-inflammatory biomarkers compared to healthy controls. Conversely, there is a growing body of research examining salivary metabolomics that may predict metabolic syndrome, type 2 diabetes, and obesity in children. A recent study of 744 children (age 11) showed that salivary levels of c-reactive protein, salivary insulin, and leptin were higher and adiponectin levels lower in obese children compared to healthy normal weight children (74). Other studies tested the applicability and reliability of using salivary glucose levels as a surrogate for blood levels (75–77). With the use of a regression equation, salivary glucose values could accurately be converted to blood glucose levels, providing patients, especially children with type 1 diabetes, with a non-invasive tool for self-monitoring (78).

Beyond assessing the nutritional and metabolic status of children, there have been a limited number of studies utilizing salivary proteins for disease detection in this population. Salivary biomarkers for familial juvenile SLE, a more aggressive form of the disease known to causes widespread tissue damage and inflammation, have been described (79). Interestingly, there have also been recent studies showing aberrant protein expression in the saliva of children affected with autism spectrum disorder. Research has shown that there are decreased levels of three proteins, statherin, histatin 1, and acidic proline-rich protein, in the saliva of autistic children compared to healthy controls (80, 81). In addition, Wormwood and colleagues have recently demonstrated that salivary proteomic analyses of children can inform caregivers about developing cognitive functions (82). The ability of saliva to provide a window into disrupted neurodevelopment holds enormous promise for the field, allowing caregivers insight into areas of the body once believed only to be accessible though costly neuroimaging (e.g., MRI, CT) or invasive procedures (e.g., cerebral spinal fluid, blood).

### Salivary Microbiomics and Metagenomics

According to the World Health Organization, infectious diseases are the leading cause of death of children and adolescents worldwide (83). Improved methods for earlier detection of infections, particularly in developing nations where blood sampling is not only invasive but also impractical, holds the potential for significantly improving outcomes. While the healthy human mouth contains as many as 500 million bacterial cells with more than 700 different colony species (84–86), it can also harbor and shed pathological infections. Upper respiratory infections, such as the influenza virus and human bocavirus HboV-1, a mild respiratory disease, can be detected in the saliva up to 1 year after primary infection (87, 88). In addition, cytomegalovirus (CMV), Epstein–Barr virus, human herpes virus (HHV) 6, and HHV7 can all be detected in human saliva (89–91). CMV, the most common cause of congenital hearing loss in children in developed countries (92), was one of the first viruses to be successfully detected through salivary analysis in the newborn (93). These initial reports showed not only that the virus was as readily detectable in saliva compared to more traditional assays using urine but also that saliva had a higher sensitivity for CMV detection compared to blood (94). Most recently, saliva has been shown to be an important biofluid for monitoring infectious Zika virus particles (95, 96). Salivary assays aimed at Zika RNA detection may prove to be a valuable tool for caregivers who are tracking exposure rates, transmission, and shedding of the virus.

In addition to the specific microbial detection, saliva also contains IgA, IgM, and IgG, which can assess immunological status and response to infection (97). For instance, studies have reported both the detection of specific antibodies to rotavirus infection in saliva (98), as well as the immunological response after administration of the vaccine (99). Similarly, rubella-specific IgM antibodies are detectable in children’s saliva (100), as are IgG levels of children who are seropositive for mumps, measles, and the herpes simplex virus (101, 102). Moreover, the hepatitis B virus, human immunodeficiency viruses, and *Salmonella typhi* can all be identified through the use of advanced technological approaches for Ig detection in the salivary fluid of children (103–105).

One of the more interesting aspects of working with saliva as a biofluid for clinical assessment is the fact that it harbors hundreds of organisms. In recent years, the field of metagenomics, defined as the genomes of all the organisms living in a specific environment in the human body, has emerged (106). Our ability to analyze not simply human gene and protein expression but also the organisms residing in the mouth that may be responsible for such a response, provides yet another opportunity to improve child health. Unique microbial colonization patterns have been shown to be associated with disease including childhood caries, NEC, and metabolic syndrome. Identifying aberrant microbial colonization patterns, while simultaneously monitoring an individual’s unique immune and inflammatory response, may allow for the development of preventative strategies to improve health outcomes (86, 107).

### Other Considerations and Applications

In 2014, the Centers for Disease Control reported that the prevalence of children ≥12 years old using illicit drug was 12% (107), and the use of non-medical psychotherapeutic drugs was 2.5%. Sadly, as drug abuse has become increasingly common, novel detection methods, including those that can be performed easily in the either home or office under direct visualization, are needed. Salivary drug screening assays have already been approved by the USFDA and are available for medical and commercial use (e.g., http://americanscreeningcorp.com). Cocaine, amphetamines, opioids, benzodiazepines, and tetra-hydro-cannabinoids, among others, can all be detected and quantified in saliva (108–111). Further, concentrations of drug metabolites of a variety of prescribed drugs are also measurable in saliva (112, 113), making ease of obtaining therapeutic levels without invasive, serial phlebotomy possible.

## Conclusion

Salivary diagnostics are primed to have an important impact on infant and child health. Whether accessing the genome, exploring real-time gene and protein expression during development, or evaluating the metabolic and infectious status of the individual, applying the latest technological advances to salivary analysis can provide valuable insight into the health of the child in a safe, non-invasive manner. However, careful attention must be made to age, diet, and developmental stage when designing assays. Establishing normative values of gene and protein expression, as well as metabolites and microbes, to account for normal variations across the age spectrum can only be achieved through prospective, large-scale, multicenter trials. Only then can data be interpreted appropriately and the hope of translating salivary diagnostic into pediatric carefully realized.

## Author Contributions

JM conceived of, wrote, and edited this review. MH wrote and edited this review. Please note: JM is the *senior* and *last* author on this manuscript.

## Conflict of Interest Statement

The authors declare that the research was conducted in the absence of any commercial or financial relationships that could be construed as a potential conflict of interest.
